# Development of ITS sequence-based markers to distinguish *Berberis aristata* DC. from *B. lycium* Royle and *B. asiatica* Roxb.

**DOI:** 10.1007/s13205-010-0001-5

**Published:** 2011-01-18

**Authors:** Subramani Paranthaman Balasubramani, Gurinder Singh Goraya, Padma Venkatasubramanian

**Affiliations:** 1 Centre for Pharmacognosy, Pharmaceutics and Pharmacology, Institute of Ayurveda and Integrative Medicine (I-AIM), Foundation for Revitalization of Local Health Traditions (FRLHT), 74/2, Jarakabande Kaval, Attur Post, Via Yelahanka, Bangalore, 560106 India; 2 Indian Forest Service, Sundernagar, Mandi, 174402 Himachal Pradesh India

**Keywords:** Daruharidra, *Berberis* spp., Quality control, Internal transcribed spacer (ITS), DNA marker

## Abstract

The stems of *Berberis aristata* DC. (Berberidaceae) are used in the South Asian traditional medicine as a key ingredient in formulations for eye care, skin diseases, jaundice, rheumatism and also in diabetes. *B. lycium* Royle and *B. asiatica* Roxb. are traded as equivalents of *B. aristata*. Conventional macro-morphology and microscopic examination does not aid in critically distinguishing the three species. DNA markers were developed by amplifying and sequencing the complete internal transcribed spacer region (ITS1, 5.8S rRNA and ITS2) from the genomic DNA, using universal primers. The markers developed are efficient and reliable in authenticating *B. aristata*, *B. asiatica* and *B. lycium*. These are useful as molecular pharmacognostic tool in quality control of raw drugs.

## Introduction

Medicinal herbs are moving from fringe to mainstream use with a greater number of people seeking remedies and health approaches free from side effects caused by synthetic chemicals (Dubey et al. 2004). With so much demand for traditional herbal medicines, it is imperative to ensure and maintain authenticity of the various botanical entities used as raw material. However, the conventional quality assurance tools sometimes have limitations in critical identification of raw materials derived from closely related species, substitutes or adulterants.

‘Daruharidra’ (in Sanskrit means ‘the wood having yellow color’) is one such botanical entity that poses problems in terms of field identification. Botanically correlated to *Berberis aristata* DC. (Berberidaceae) it has been used in Ayurveda and Traditional Chinese Medicine for the past 3,000 years (Anonymous 1999; Sack and Froehlich 1982). In Ayurveda, it forms a key ingredient in preparation of formulations for eye care, wounds, skin diseases, jaundice, rheumatism and also in diabetes (Anonymous 1999). The genus *Berberis* constitutes of spinous shrubs distributed throughout the Himalayas, from Bhutan to Kunawar (altitude 6,000–10,000 ft) in India and Sri Lanka (altitude 6,000–7,000 ft) (Kirtikar and Basu 1995). *B. aristata* is a plant species of high trade sourced from temperate forests. The present annual production and supply of *B. aristata* in India is dependent only from the state of Himachal Pradesh (Ved and Goraya 2008).

In field, more than one species of *Berberis* usually occur together. The nature of the *Berberis* species varies with place and the leaves exhibit great variations (Rao et al. 1998). So, Rao et al. (1999) reported pollen morphologic studies in solving taxonomic problem in this complex group. Since harvesting of *Berberis* spp. takes place during winter months when the key phenological features for identification are not present, it becomes difficult to distinguish one species from the other in the field. Thus, species other than *B. aristata* are also harvested. Since stems are the parts used it becomes even more challenging to obtain authentic collections and assure quality. As a result, *B. asiatica* Roxb. and *B. lycium* Royle are traded and used as equivalents/substitutes of *B. aristata* (Anonymous 2003; Ved and Goraya 2008). Conventional Pharmacognosy techniques based on macro-morphological characters may not be effective in distinguishing the three species traded as ‘Daruharidra’. Further, related species may share similar histological characteristics, making microscopic examination not so accurate (Yan-Bo et al. 2007).

Different species of the same genus may have totally different or weaker pharmacological action as compared with the authentic counterpart (Yip et al. 2007). Therefore, more objective and definitive methods are necessary for identification and authentication of herbs. Extracts of *B. aristata* are said to possess anti-amebic (Sohni et al. 1995), anti-microbial (Singh et al. 2007), wound healing (Biswas and Mukherjee 2003), anti-oxidant and anti-hyperglycemic properties (Singh and Kakkar 2009). *B. asiatica* has been reported to have anti-microbial and anti-tumor effects (Kumar et al. 1998; Singh et al. 2007) while *B. lycium* is reported to possess anti-bacterial, anti-diabetic effect and is also used to treat bleeding piles (Singh et al. 2007; Ahmad and Alamgeer 2009).

Berberine, an alkaloid is considered to be the active compound present in *Berberis* spp*.* with diverse pharmacological effects and is also considered as a nutraceutical (Saied et al. 2007; Wongbutdee 2009; Affuso et al. 2009)*.* Since, berberine is present in all the three species of *Berberis,* i.e., *B. aristata*, *B. lycium* and *B. asiatica*, with inherent inter and intra species quantitative differences, phytochemical profiling may not reflect the exact identity of the raw drug (Chandraa and Purohita 1980; Srivastava et al. 2004). The annual trade of Daruharidra including the substitutes is estimated to be 500–1,000 Metric Tonnes (Ved and Goraya 2008). According to the WHO general guidelines for methodologies on research and evaluation of traditional medicines, first step in assuring quality, safety and efficacy of traditional medicines is correct identification (World Health Organization 2000).

DNA-based markers have now become a popular means for the identification and authentification of plants because genetic composition is unique for each individual irrespective of the physical form and is less affected by age, physiological condition, environmental factors, harvest, storage and processing. DNA extracted from leaves, stems or roots of a herb all carry the same genetic information and extracted DNA can be stored for longer duration as they are stable (Yip et al. 2007). Nuclear ribosomal RNA genes and internal transcribed spacer (ITS) sequences have become favored markers in evolutionary studies at different taxonomic levels. In recent days, these sequence variations are used to develop specific markers for the identification and authentication of raw drugs and herbal formulations (Yan-Bo et al. 2007; Balasubramani et al. 2010).

The objective of the present investigation was to develop molecular markers for distinguishing *B. aristata*, *B. asiatica* and *B. lycium* based on their nuclear DNA ITS sequence.

## Materials and methods

### Plant material

Field samples of *B. aristata, B. asiatica* and *B. lycium,* were collected from different geographical locations of Himachal Pradesh in India. The authenticity of the samples was confirmed by qualified taxonomist. Each sample was assigned a specific laboratory identification number as indicated in Table 1. Voucher specimens were deposited in the Herbarium and Raw Drug Repository (FRLH), Bangalore, India.

**Table 1:** Details of the *Berberis* sp. stem samples used in the study

Si no.	Name of the species	Place of collection	Accession No.
1	*B. aristata*	Taradevi forest, Shimla	L/06/10/010
2		Taradevi forest, Shimla	L/06/10/011
3		Potter’s hill, Shimla	L/08/09/012
4		Shillaru, Shimla	L/08/10/008
5		Potter’s Hills, Shimla	L/08/10/011
6	*B. asiatica*	Mulshi, Mussoorie	L/07/12/001
7		Urla village, Mandi Dt.	L/08/03/001
8		Saharan-Nahan Road	L/08/09/013
9		Saharan-Nahan road	L/08/09/016
10	*B. lycium*	Karol, Manikaran, Kullu Dt.	L/06/06/037
11		Kangra, Palmpur	L/06/10/008
12		Potter’s hills, Shimla	L/08/09/014
13		Shillaru, Kangra	L/08/10/009
14		Chail, Solan Dt.	L/08/10/010

The accessions were collected from different locations of Himachal Pradesh, India (HP-latitude 32°29′N; longitude 75°10′E)

### Chemicals

Tris–HCl, EDTA, NaCl, Cethyltrimethylammonium bromide (CTAB), Polyvinyl pyrrolidone (PVP), β mercaptoethanol, isopropanol, agarose, boric acid, and ethanol were purchased from Sigma Chemicals (Sigma-Aldrich, Bangalore, India). Enzymes (*Taq* Polymerase and RNase A), buffer, MgCl_2_ and dNTPs for PCR amplification were purchased from Bangalore Genei (Bangalore, India).

### Genomic DNA extraction

Stem samples of *B. aristata, B. asiatica* and *B. lycium* were cut into small pieces and dried in a dehydrator at 48–50 °C (Hardfacts, Mumbai, India) until a constant dry weight is obtained. The dried stem pieces were stored at room temperature until use. Total genomic DNA was extracted following the protocol described by Milligan (1998) with modifications. The dried stem pieces were ground into coarse powder in a domestic blender. Approximately, 100 mg of the coarse powder was ground with liquid nitrogen to a fine powder. Extraction buffer containing 2% (w/v) CTAB, 1.4 M NaCl, 20 mM EDTA, 100 mM Tris–HCl (pH 8), 1% (w/v) PVP and 0.2% (v/v) β mercaptoethanol (pH 7.5–8.0) was added to the powder. The slurry was incubated at 60 °C in a water bath for 60–90 min followed by extraction using chloroform:isoamyl alcohol (24:1) (v/v) with centrifugation at 9,000 rpm for 10 min at 4 °C. The aqueous phase was collected and the extraction process was repeated twice. Finally, the DNA was precipitated from pooled aqueous phase using ice cold ethanol. Nucleic acid was recovered by centrifugation at 8,000 rpm for 15 min at 4 °C and the pellet was dissolved in TE buffer (10 mM Tris–Hcl, 1 mM EDTA; pH 8). The contaminating RNA was removed by treating with RNase A (20 μg/μL) for 30 min at 37 °C. The quantity and purity of DNA were checked using UV spectrophotometer by calculating the *A*_260_/*A*_280_ ratio (Shimadzu, Tokyo, Japan) (Fai and Ngan 2002). The DNA stock concentration was maintained at 30–50 ng/μL.

### Amplification of ITS region

Double-stranded DNA of the complete ITS region (ITS1, 5.8S rRNA gene and ITS2) of the test species was amplified with the universal primers ITS5 (forward primer; 5′ GGAAGTAAAAGTCGTAACAAGG 3′) and ITS4 (reverse primer; 5′ TCCTCCGCTTATTGATATGC 3′) (White et al. 1990). The primers were custom synthesized by Bioserve biotechnologies (Hyderabad, India). Amplification was carried out in 25 μL reaction volume with sterile distilled water, 2.5 μL 10× *Taq* buffer, 2.0 mM MgCl_2_, 0.6 mM dNTP mixture, 30 pM of each primer, 1.5 U *Taq* DNA polymerase and approximately 60 ng of genomic DNA. PCR was performed in an Eppendorf Mastercycler Gradient (Hamburg, Germany). The amplification profile was 94 °C for 4 min followed by 35 cycles of 94 °C for 80 s, 60 °C for 40 s, 72 °C for 80 s with a final extension step at 72 °C for 4 min.

PCR products were resolved on 1.2% agarose, 0.5× TBE gels pre-stained with ethidium bromide (0.5 μg/ml). Simultaneously, 100 bp DNA ladder (Fermentas, Ontario, Canada) was loaded to confirm the size of the amplicon. The gel was visualized under UV radiation in a Camag Reprostar (Camag, Muttenz, Switzerland) and image captured (Canon, Tokyo, Japan). Each experiment was repeated at least three times with all available accessions (Table 1) of each plant species to confirm its reproducibility and repeatability.

### Direct sequencing of complete ITS region

PCR amplified complete ITS region of *B. aristata, B. asiatica* and *B. lycium* were purified from agarose gel using QIAquick gel extraction kit (Qiagen, Maryland, USA) following manufacturer’s instruction. Direct sequencing of the amplicon was done using primers ITS5 and ITS4 in an automated ABI 3100 Genetic Analyser (Applied Biosystems, CA, USA) by Bangalore Genei (Bangalore, India).

### Sequence analysis and development of species-specific markers for *B. aristata, B. asiatica* and *B. lycium*

Sequence boundaries of the ITS1, 5.8S and ITS2 were determined following the submitted sequence data available on GenBank (http://www.ncbi.nlm.nih.gov/). The complete sequence of the ITS region for each species studied was then deposited at GenBank. Multiple alignment was done with complete ITS sequences of *B. aristata, B. asiatica* and *B. lycium,* employing CLUSTALW (http://www.ebi.ac.uk.clustalw/). Homology percentage was calculated using the following formula:

$$ {\text{Homology}}\;\% = {\frac{\text{Number of homologous bases in the species compared}}{\text{Total Number of bases in the species compared with}}} \times 100 $$

DNA primers capable of giving specific amplification with individual species were developed using NCBI primer blast tool (http://www.ncbi.nlm.nih.gov/tools/primer-blast/). The oligonucleotides were custom synthesized by Bioserve biotechnologies (Hyderabad, India). PCR reaction with the species-specific primer set contained 30 ng of genomic DNA, 2.5 μl of 10× assay buffer, 1.5 mM MgCl_2_, 0.3 mM dNTP mix, 30 pM of each primer and 1 U of *Taq* DNA polymerase (Bangalore Genei, India) with the volume made up to 25 μl with sterile distilled water. PCR program conditions optimized for the species-specific primer sets were as follows: 94 °C for 4 min, followed by 35 cycles of 35 s at 94 °C, 30 s at 60 °C and 45 s at 72 °C with a final extension step for 3 min at 72 °C.

## Results

### Extraction of genomic DNA and amplification of complete ITS sequence

**Fig. 1:**
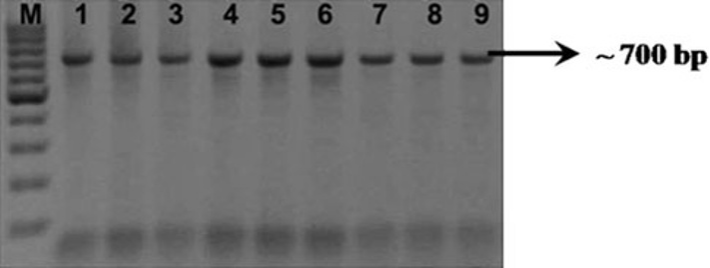
PCR amplification of complete ITS sequence. *Lane M* 100 bp DNA ladder; *lanes 1–3 B. aristata*; *lanes 4–6 B. asiatica*; *lanes 7–9 B. lycium*. Approximately, 700 bp amplicon can be observed with all samples of *Berberis* spp.

After trying with many reported DNA extraction protocols, the method described in “Materials and methods” section repeatedly yielded good quality, high molecular weight genomic DNA from the dried stem samples of *B. aristata*, *B. asiatica* and *B. lycium*. The procedure yielded 400–600 ng of DNA per 100 mg of tissue. An absorbance (*A*_260_/*A*_280_) ratio of 1.6–1.8 indicated insignificant levels of contaminating proteins and polysaccharides. The universal primers ITS5 (forward) and ITS4 (reverse) amplified the complete ITS region (ITS1, 5.8S rRNA gene and ITS2) yielding an amplicon of approximately 700 bp for all the three species studied (Fig. 1).

### Sequencing of complete ITS region and homology analysis

**Fig. 2:**
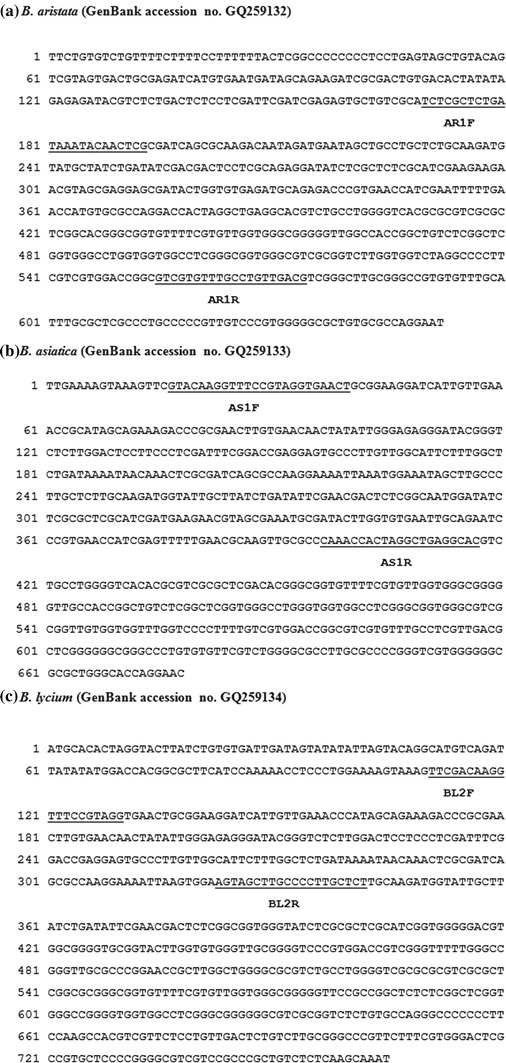
Complete ITS sequence of *B. aristata*, *B. asiatica* and *B. lycium.* Species-specific 20 mer primers (forward and reverse) for each species is shown underlined in the sequence

**Fig. 3:**
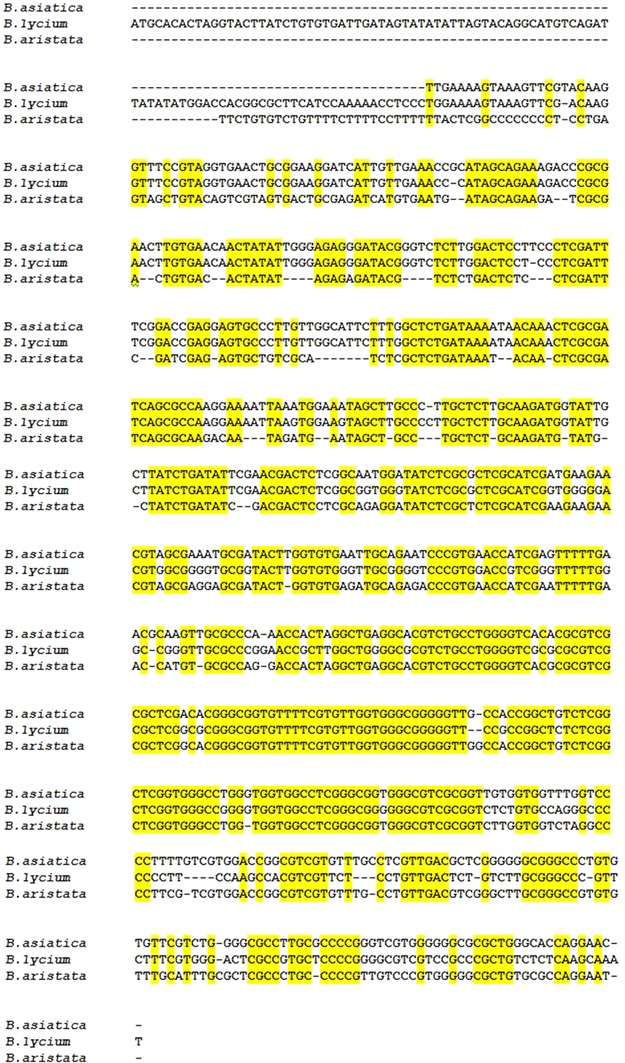
Multiple alignment of the complete ITS sequence of *B. aristata*, *B. asiatica* and *B. lycium*

Direct sequencing of the gel purified amplicon yielded a 652 bp sequence for *B. aristata* (GeneBank accession no. GQ259132) (Fig. 2a), 678 bp for *B. asiatica* (GeneBank accession no. GQ259133) (Fig. 2b) and 765 bp for *B. lycium* (GeneBank accession no. GQ259134) (Fig. 2c). BLAST analysis indicated that the sequences were novel. Multiple alignment of rRNA and ITS sequence of *Berberis* spp. under study revealed inter-species sequence variation (Fig. 3). The homology score in the ITS1, 5.8S rRNA gene and ITS2 regions of *B. aristata*, *B. asiatica* and *B. lycium* are shown in Table 2. Interestingly, the data from sequence alignment indicated that the ITS1, 5.8S rRNA gene and ITS2 regions of each species were quite unique and different from each other. Maximum homology was observed in the ITS1 regions of *B. asiatica* and *B. lycium* (97.86%) while lowest (56.41%) was seen in the ITS1 regions of *B. asiatica* and *B. aristata* (Table 2). The sequence variation was used to develop species-specific markers for individual species.

**Table 2:** The Homology Score in the ITS Region of *B. aristata, B. asiatica and B. lycium*

	*B. aristata*			*B. asiatica*			*B. lycium*		
	ITS1	5.8S	ITS2	ITS1	5.8S	ITS2	ITS1	5.8S	ITS2
*B. aristata*									
ITS1	**–**	**–**	**–**	67.00%	**–**	**–**	72.08%	**–**	**–**
5.8S	**–**	**–**	**–**	**–**	82.89%	**–**	**–**	72.36%	**–**
ITS2	**–**	**–**	**–**	**–**	**–**	84.86%	**–**	**–**	80.27%
*B. asiatica*									
ITS1	56.41%	**–**	**–**	**–**	**–**	**–**	97.86%	**–**	**–**
5.8S	**–**	83.56%	**–**	**–**	**–**	**–**	**–**	78.76%	**–**
ITS2	**–**	**–**	86.72%	**–**	**–**	**–**	**–**	**–**	74.88%
*B. lycium*									
ITS1	60.94%	**–**	**–**	99.14%	**–**	**–**	**–**	**–**	**–**
5.8S	**–**	75.34%	**–**	**–**	79.45%	**–**	**–**	**–**	**–**
ITS2	**–**	**–**	78.44%	**–**	**–**	72.47%	**–**	**–**	**–**

### Designing of species-specific markers and validation

**Fig. 4:**
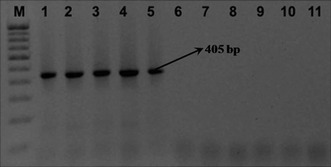
PCR amplification of genomic DNA from *Berberis* spp. with AR1F and AR1R. *Lane M* 100 bp DNA ladder; *lanes 1–5 B. aristata*; *lanes 6–8 B. asiatica*; *lanes 9–11 B. lycium*. A 405 bp amplicon is observed only in *B. aristata* samples

**Fig. 5:**
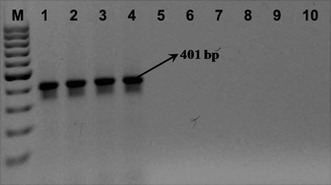
PCR amplification of genomic DNA from *Berberis* spp. with AS1F and AS1R. *Lane M* 100 bp DNA ladder; *lanes 1–4 B. asiatica*; *lanes 5–7 B. lycium*; *lanes 8–10 B. aristata*. A 401 bp amplicon is observed only in *B. asiatica* samples

**Fig. 6:**
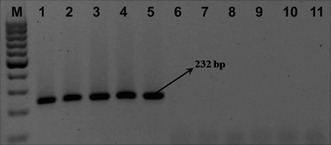
PCR amplification of genomic DNA from *Berberis* spp. with BL2F and BL2R. *Lane M* 100 bp DNA ladder; *lanes 1–5B. lycium*; *lanes 6–8 B. aristata*; *lanes 9–11 B. asiatica*. A 232 bp amplicon is observed only in *B. lycium* samples

NCBI primer blast tool was used to develop and validate the species-specific markers. The details of species-specific primers designed and amplicon size are given in Table 3. The *B. aristata*-specific primers (AR1F and AR1R) yielded 405 bp amplicon with all the accessions of *B. aristata* only and did not produce any amplification with *B. asiatica* and *B. lycium* (Fig. 4). Similarly, primers AS1F and AS1R produced amplification (401 bp) only with *B. asiatica* accessions (Fig. 5), while BL2F and BL2R primers yielded 232 bp amplicon only with *B. lycium* samples (Fig. 6).

**Table 3:** Details of the species-specific markers designed using the ITS sequence

Plant species	Name of the marker	Sequence of the DNA marker (5′→ 3′)	Product (bp) size
*B. aristata*	AR1F	TCTCGCTCTGATAAATACAACTCG	405
	AR1R	CGTCAACAGGCAAACACGAC	
*B. asiatica*	AS1F	GTACAAGGTTTCCGTAGGTGAACT	401
	AS1R	GTGCCTCAGCCTAGTGGTTTG	
*B. lycium*	BL2F	TTCGACAAGGTTTCCGTAGG	232
	BL2R	AGAGCAAGGGGCAAGCTACT

## Discussion

One of the impediments in the acceptance of herbal formulations is the lack of standardization and quality control profiles. Due to the complex nature and inherent variability of the chemical constituents of plant-based drugs, it is difficult to establish quality control parameters using phytochemical tools (World Health Organization 2000). The pitfalls associated with the common conventional Pharmacognosy methods like macro-microscopic examination and phytochemical analysis have led to the search of definitive methods for authentication of herbs and formulations (Yan-Bo et al. 2007). Chinese pharmacopoeia has already started including modern DNA-based details to distinguish herbs (Yip et al. 2007).

Our experience with *B.aristata, B. asiatica* and *B. lycium* indicates that they can hardly be distinguished based on their morphology and histology (data unpublished). Controversial reports on the berberine content of these three species have been reported in the past. A comparative high performance thin layer chromatography (HPTLC) analysis of *B. asiatica* with *B. aristata* showed identical profile with lesser berberine content in the former (Srivastava et al. 2004). While, Andola et al. (2010) have reported that *B. asiatica* has higher berberine content followed by *B. lycium* and *B. asiatica*. These inconsistent reports indicate the disadvantage of chemical marker and also insist on a robust technique for differentiating these three species.

The commonly followed DNA fingerprinting techniques like randomly amplified polymorphic DNA (RAPD), restriction fragment length polymorphism (RFLP) and amplified fragment length polymorphism (AFLP) have poor reproducibility of markers (Yan-Bo et al. 2007). Recently, more reproducible sequence characterized amplified region (SCAR) markers have been developed for many medicinal plant species to distinguish from their substitutes or adulterants (Devaiah and Venkatasubramanian 2008; Devaiah et al. 2010). Many studies in recent years have employed ITS sequences as genetic markers for various species at generic and intrageneric levels. The length and sequences of ITS regions of ribosomal RNA gene repeats are believed to rapidly turn over thus capturing measurable variations between species (Dubouzet and Shinoda 1999). Universal PCR primers designed from highly conserved regions flanking the ITS region, relatively small in size (600–700 bp) and high copy number (>100 per cell), enable easy amplification of the ITS region (Yan-Bo et al. 2007; Yip et al. 2007). These advantages have made ITS region and ribosomal RNA gene sequence as preferred choice for molecular typing. Novak et al. (2007) have reported ITS sequence-based authentication of *Echinacea* spp. in extracts. Balasubramani et al. (2010) have reported ITS sequence-based DNA markers to differentiate *Tribulus* spp*.* of the Zygophyllaceae family. Watthanachaiyingcharoen et al. (2010) have reported 18S rRNA gene-based PCR–RFLP to distinguish *Coscinium fenestratum* from other genus in the Menispermaceae family.

The main problem with DNA-based methods for identification is the presence of phenolic compounds, acidic polysaccharides and pigments that hinder the PCR amplification. This can be avoided by choosing the most suitable DNA extraction process that helps to eliminate the PCR inhibitors. DNA-based methods have been successfully used to even authenticate herbal constituents in commercial herbal preparations in which the components have been ground, boiled, concentrated, dried and blended (Yip et al. 2007). DNA information may not be directly correlated with the amounts of the active ingredients and this is one disadvantage.

In this study, we have described ITS sequence-based efficient and reliable DNA markers to identify and distinguish *B. aristata*, *B. asiatica* and *B. lycium*. These markers can be used as a molecular pharmacognostic tool for quality control of herbal raw drugs.
